# Disclosing Bias in Bisulfite Assay: MethPrimers Underestimate High DNA Methylation

**DOI:** 10.1371/journal.pone.0118318

**Published:** 2015-02-18

**Authors:** Andrea Fuso, Giampiero Ferraguti, Sigfrido Scarpa, Isidre Ferrer, Marco Lucarelli

**Affiliations:** 1 Dept. of Psychology, Sapienza University of Rome, Rome, Italy; 2 European Center for Brain Research (CERC)/IRCCS Santa Lucia Foundation, Rome, Italy; 3 Dept. of Cellular Biotechnologies and Hematology, Sapienza University of Rome, Rome, Italy; 4 Dept. of Surgery “P. Valdoni”, Sapienza University of Rome, Rome, Italy; 5 Institute of Neuropathology, IDIBELL-Bellvitge University Hospital and University of Barcelona, CIBERNED, L'Hospitalet de Llobregat, Spain; 6 Pasteur Institute, Cenci Bolognetti Foundation, Sapienza University of Rome, Rome, Italy; Università di Napoli Federico II, ITALY

## Abstract

Discordant results obtained in bisulfite assays using MethPrimers (PCR primers designed using MethPrimer software or assuming that non-CpGs cytosines are non methylated) versus primers insensitive to cytosine methylation lead us to hypothesize a technical bias. We therefore used the two kinds of primers to study different experimental models and methylation statuses. We demonstrated that MethPrimers negatively select hypermethylated DNA sequences in the PCR step of the bisulfite assay, resulting in CpG methylation underestimation and non-CpG methylation masking, failing to evidence differential methylation statuses. We also describe the characteristics of “Methylation-Insensitive Primers” (MIPs), having degenerated bases (G/A) to cope with the uncertain C/U conversion. As CpG and non-CpG DNA methylation patterns are largely variable depending on the species, developmental stage, tissue and cell type, a variable extent of the bias is expected. The more the methylome is methylated, the greater is the extent of the bias, with a prevalent effect of non-CpG methylation. These findings suggest a revision of several DNA methylation patterns so far documented and also point out the necessity of applying unbiased analyses to the increasing number of epigenomic studies.

## Introduction

It is generally accepted that DNA methylation almost exclusively occurs in CpG dinucleotides in mammals [1–4]. Non-CpG methylation has been documented, but with limited extent (see Discussion) and in specific cell types—mainly stem cells. Using the bisulfite modification followed by PCR amplification, cloning and sequencing, we previously reported unexpectedly high non-CpG methylation in *myogenin* mouse promoter [5]. Moreover, in human *PSEN1* promoter, we observed discordant methylation patterns when using PCR primers designed using the MethPrimer software [6] (assuming that non-CpG cytosines are modified by bisulfite; defined as “MethPrimers” from now on) or primers designed to be insensitive to cytosine-methylation status. When the bisulfite technique has been originally described [7, 8], it was recommended not to include cytosines in the recognition sequence of the primers to avoid possible mismatches depending on methylation status. Interestingly, the same authors were able to evidence some non-CpG methylation in the first applications of the technique [9]. However, in the following years, MethPrimers became the most (if not exclusively) used primers in bisulfite-based applications, due to the advantage of the software-assisted primer design and to the general assumption that non-CpG cytosines were mainly unmethylated. Although the lack of a specific name makes it difficult to retrieve in PubMed the number of papers in which they are used, looking at the citations of the original article describing the MethPrimer software [6], it is possible to infer at least one thousand citations. Furthermore, an on line search through Google Scholar evidences about 29300 articles in which the bisulfite approach is used; among these, about 80% reports the use of MethPrimer software or similar primer design strategy.

Despite this trend, we have always been using primers designed in regions without cytosines or, when this was not possible, primers with degenerated bases (G/A) to cope with the uncertain C/U conversion of the few (max. 3) cytosines residues included in the sequence of the primer [5,10]. These primers will be here defined as “methylation-insensitive primers” (MIPs). The high non-CpG methylation observed for *myogenin* and the discordant methylation profile observed when *PSEN1* was analyzed using either MethPrimers or MIPs lead us to hypothesize that MethPrimers could negatively select non-CpG methylated DNA molecules also resulting in a biased outcome of the CpG methylation assessment.

In order to verify this hypothesis, we analyzed two genes (*myogenin* and *PSEN1*), each one in two experimental models: CD1 mouse embryos and C2C12 myoblast cells for *myogenin* and human brains and neuroblastoma SK-N-BE cells for *PSEN1*. In each model, we compared conditions with differential methylation profiles, as ascertained in previous studies [5, 10].

## Results and Discussion

Samples were bisulfite-modified and then amplified by two primer sets, MethPrimers or MIPs, recognizing the same promoter region (S1 Fig.); each sample was divided in two aliquots after bisulfite conversion for amplification with the two primer sets and then processed in parallel until the final sequencing step. Several positive and negative controls were performed to avoid any possible technical bias (see Methods section).

**Fig 1:**
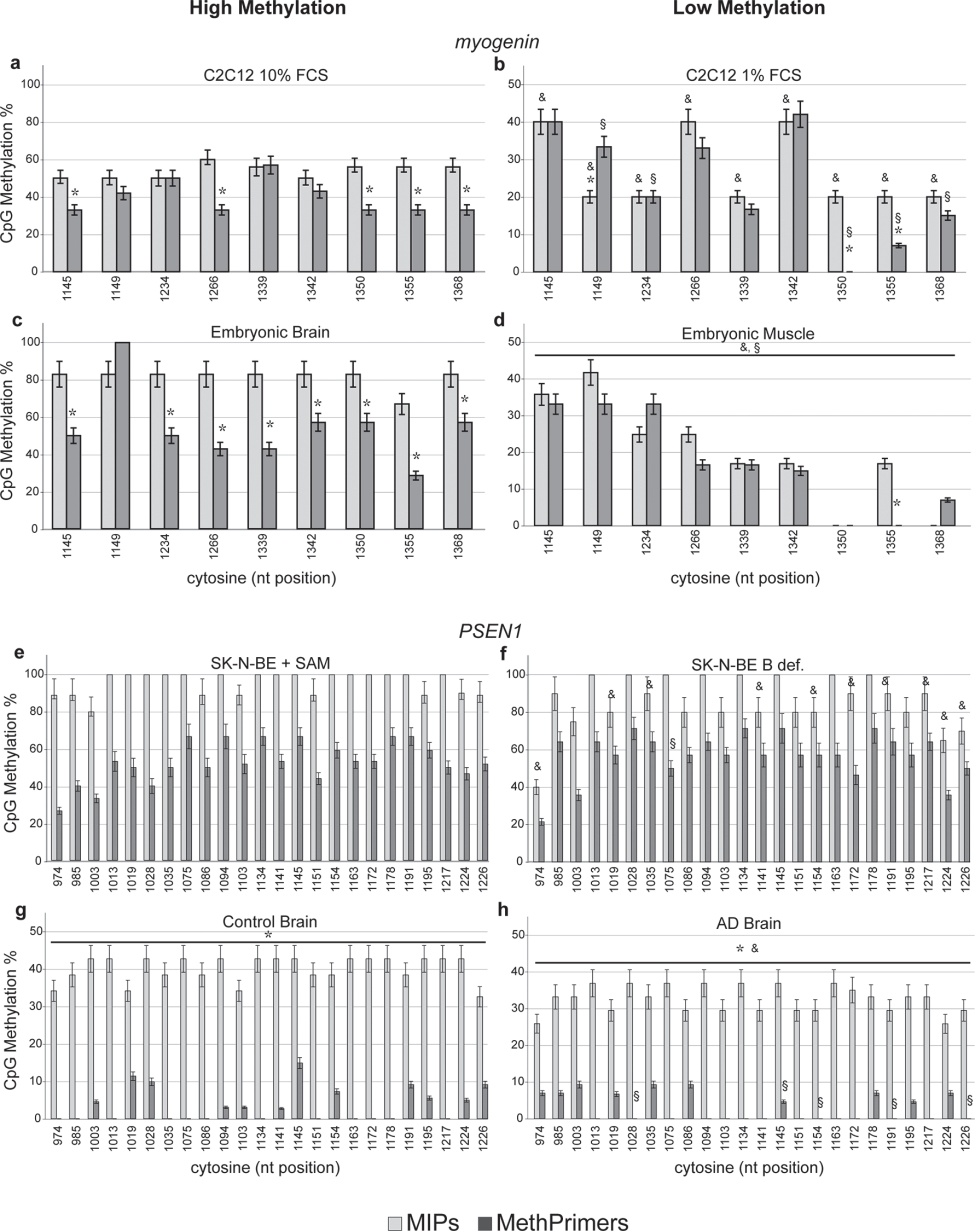
MIPs and MethPrimers result in different CpG methylation patterns. CpG methylation pattern is expressed as percent methylation for each CpG in the investigated region of the mouse *myogenin* (a-d) and human *PSEN1* (e-h) promoters. Light grey columns show the values obtained using MIPs, dark grey columns show the values obtained using MethPrimers. Time points for cell cultures are: 48 h for C2C12 10% FCS, SK-N-BE+SAM, SK-N-BE B def.; 24 h for C2C12 1%FCS. Symbols: * *p*<0.05 MIPs vs. MethPrimers; &: *p*<0.05 High Methylated vs. Low Methylated with MIPs; §: *p*<0.05 High Methylated vs. Low Methylated with MethPrimers. Y axes in histograms have a different scale (up to 100% for high methylated samples, up to 50% for low methylated samples) in order to better evidence intra-sequence differences in cytosine methylation analyzed with Methprimers vs. MIPs.

Fig. 1 shows the methylation pattern of the 9 CpG sites investigated in *myogenin* promoter in C2C12 cells (Fig. 1A and 1B) and in mice tissues (Fig. 1C and 1D). When *myogenin* methylation is high as in cells grown in 10% FCS and in mouse embryonic brain, MethPrimers significantly underestimate DNA methylation levels. As a matter of fact, whereas Mann-Witney test (used to evidence differences between two samples analyzed with the same primers) results in a significant difference for all the cytosine moieties when comparing the hypermethylated (Fig. 1A and 1C) vs. hypomethylated (Fig. 1B and 1D) experimental condition using MIPs [Cells: U = 9.00, *p*<0.05; Tissues: U = 16.00, *p*<0.02], the same comparison using MethPrimers indicates non-significant differences for 4 cytosines (1145, 1266, 1339, 1342). Moreover, Wilcoxon test (used to evidence differences when comparing MIPs vs. MethPrimers assay in the same sample) indicates that 5 cytosines result significantly hypomethylated when analyzed with MethPrimers [cytosines 1145, 1266, 1350, 1355, 1368: Z = -1.6, *p*<0.05] in C2C12 myoblasts. This difference was much more evident in brain and muscle tissues: all the cytosines (except the 1149) resulted significantly hypomethylated when the methylation assay is performed using MethPrimers vs. MIPs in the most methylated condition [Z = -2.5, *p*<0.02]. It is therefore evident that in experimental conditions in which DNA methylation is high MethPrimers show low power of detection compared to MIPs, eventually failing to evidence differences between differential methylation in two samples.

Very similar results were obtained when studying DNA methylation pattern of *PSEN1* in SK-N-BE cells grown in hypermethylating (S-adenosylmethionine supplemented) or hypomethylating (B vitamin deficiency) conditions [11] (Fig. 1E and F) and in frontal cortex samples from control subjects and patients with Alzheimer’s disease (AD) and control subjects (Fig. 1G and H). Mann-Withney test resulted in 10 out of 24 cytosines moieties significantly hypomethylated in *PSEN1* promoter of low methylated (Fig. 1F) vs. high methylated (Fig. 1E) SK-N-BE cells when MIPs were used [cytosines 974, 1019, 1035, 1141, 1154, 1172, 1191, 1217, 1224, 1226: U = 9.00, *p*<0.05]. On the contrary, only 1 cytosine resulted significantly hypomethylated in the same samples when MethPrimers were used [cytosines 1075: U = 9.00; *p*<0.05]. This result was, also for PSEN1, more evident in tissues: all the cytosines analyzed were significantly hypomethylated in AD brains vs. controls [U = 16.00, *p*<0.05] whereas only 5 cytosines resulted hypomethylated when studied using MethPrimers [cytosines 1028, 1145, 1154, 1191, 1226: U = 16.00, *p*<0.05]. The inter-assay variation was confirmed by Wilcoxon test also for *PSEN1*; as a matter of fact, all the cytosines resulted significantly hypomethylated when the assay was performed using MethPrimers vs. MIPs [SK-N-BE cells: Z = -2.52, *p*<0.05; brain: Z = -2.52, *p*<0.02]. Therefore, assessing methylation with MethPrimers can result in underestimation of the high methylation levels eventually biasing the detection of differences.

The Sanger sequencing after bisulfite modification allows detecting the methylation level of any individual cytosine in the whole amplified region, including non-CpG cytosines [7, 8]. As previously observed for *myogenin* promoter [5] (S2 Fig.), when sequencing PCR products amplified using MIPs we were able at evidencing discrete non-CpG methylation also in *PSEN1* promoter (S3 Fig.) at least in the conditions of high methylation [*myogenin*: Z = -2.91, *p*<0.01; *PSEN1*: Z = -3.13, *p*<0.01]. It is worth of note that, whereas MIPs allow discriminating between conditions with high/discrete and low/absent non-CpG methylation, MethPrimers invariably fail to evidence it (S2 and S3 Figs.). This observation raises two considerations: i) from the technical point of view it is evident that the described underestimation of DNA methylation when MethPrimers are used is due to the inability of these primers to bind non-CpG-methylated sequences; ii) from a perspective point of view, it can be inferred that the use of these primers has been causing general underestimation of the non-CpG methylation.

**Fig 2:**
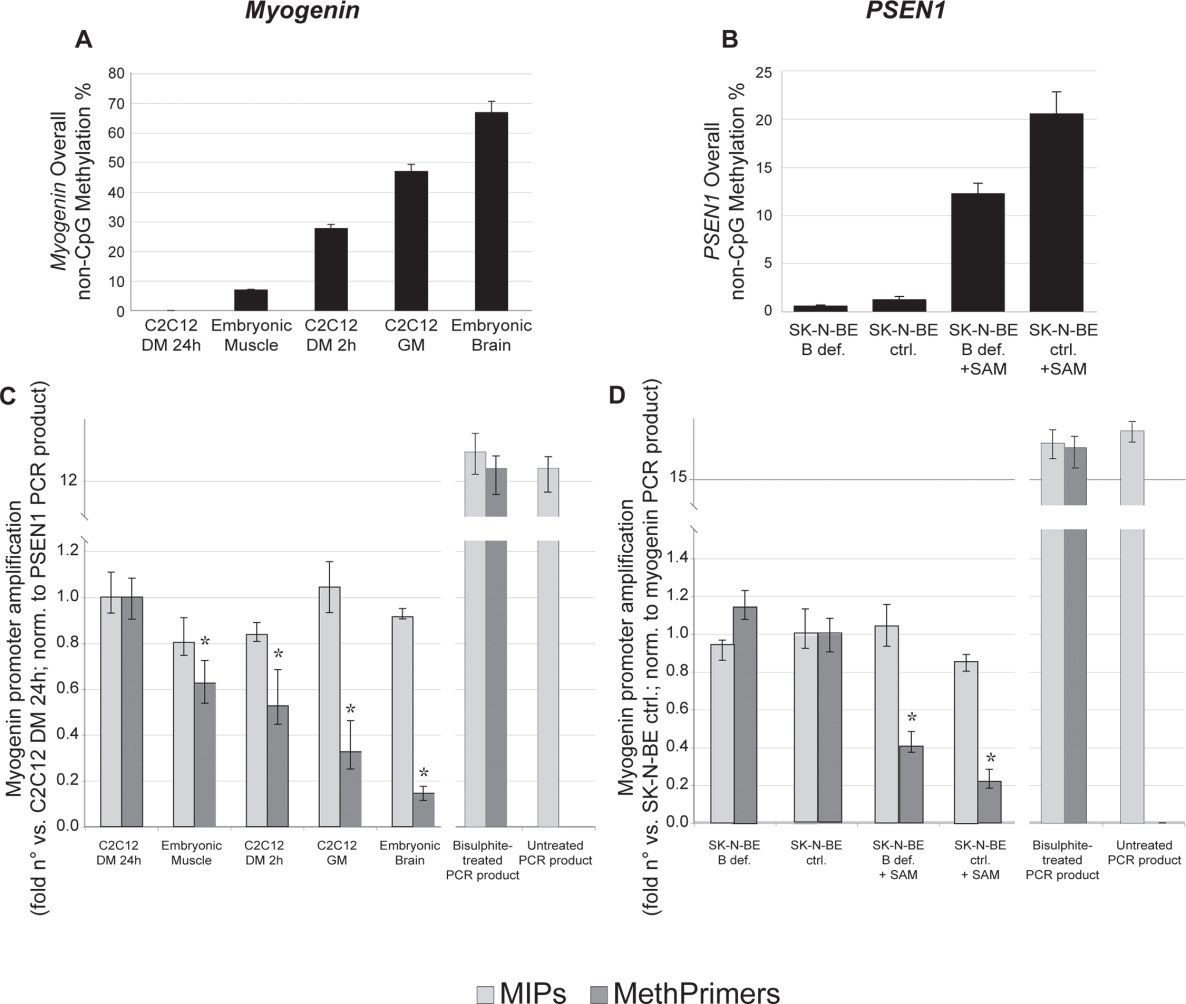
MethPrimers amplification efficiency is affected by DNA methylation. a) *Myogenin* and b) *PSEN1* overall non-CpG methylation. c) and d) Amplification efficiency of primers on samples with differential overall non-CpG methylation was assessed by Real-Time PCR assay using MIPs (light grey columns) or MethPrimers (dark grey columns) for *myogenin* (c) and *PSEN1* (d). Bisulphite-treated PCR products and untreated PCR products were used as control of amplification efficiency. Values are expressed as fold n° versus control (C2C12 in 1% 24 hours for *myogenin*, SK-N-BE in complete medium for *PSEN1 used as respective calibrators*); *myogenin* amplification values were normalized using *PSEN1* PCR product amplification (added to mouse samples and used as exogenous reference) whereas *PSEN1* amplification values were normalized to *myogenin* PCR product amplification (in this case added to human samples and used as exogenous reference). *: *p*<0.001 vs. ctrl.

In order to confirm these observations by an independent approach, we also compared, by real-time PCR on bisulphite-modified genomic DNA, the amplification efficiency of each primer set respect to differential methylation statuses (Fig. 2). Fig. 2A and 2B show the overall non-CpG methylation levels measured, respectively, for *myogenin* and *PSEN1* promoter, in different experimental conditions, ordered according to increasing methylation. Fig. 2C and 2D demonstrate that whereas *myogenin* and *PSEN1* MIPs correctly amplify with similar efficiency all bisulfite-modified DNA samples independently on their original non-CpG methylation status (light gray columns), MethPrimers show significant inverse correlation [*myogenin*: r = 0.94, *p*<0.001; *PSEN1*: r = 0.89, *p<*0.001] between amplification efficiency and DNA methylation level. This result indicates that the more DNA is non-CpG methylated, the less MethPrimers are able to bind to the bisulfite-modified product indicating that these primers negatively select the high methylation (poorly modified) DNA fraction in the sample. As expected, MethPrimers failed to amplify untreated PCR products whereas MIPs show similar amplification efficiency for both treated and untreated PCR fragments.

Finally, we used a third independent experimental approach to demonstrate that non-CpG methylation was present in the samples we analyzed. To this end, DNA samples with differential methylation levels were digested by non-CpG methylation-sensitive and methylation-insensitive endonucleases. Endonuclease assays for *myogenin* were already published [5], whereas the assay on *PSEN1* is shown in S4 Fig. The results also indicate that the cutting efficiency is impaired in DNA samples with high methylation.

These experiments show that MethPrimers, commonly used in the PCR step of several bisulfite-based assays for the study of DNA methylation, can give false negative results (no methylation differences) due to their selective binding to the DNA fraction with lower methylation. This problem particularly affects highly methylated samples whereas samples with low methylation are unaffected; the use of MIPs allows bypassing this deficiency. We do not affirm that all the data so far obtained using MethPrimers are biased, but suggest being more cautious when “no differences” in methylation patterns are found. It is likely that a number of (mainly negative) findings obtained with MethPrimers could benefit from a verification using MIPs. These data were obtained for two independent genes, in two different organisms, each one in two experimental models; this approach appears sufficient to sustain the disclosure of the technical bias. A genome-wide confrontation between MIPs and MethPrimers appears beyond the scope of this technical report and could be probably not resolutive, since we demonstrated that the bias is evident only in conditions of high DNA methylation. However, the comparison of the epigenome as evaluated by the two kinds of primers would be a natural prosecution of the experiments here reported.

Finally, although the aim of this paper is not to evidence discrete non-CpG methylation in differentiated cells, we cannot ignore that our data strongly point out the idea that non-CpG methylation extent and role are probably underestimated. Indeed, recent evidences using unbiased techniques like Whole Genome Bisulphite Sequencing (WGBS) stress a functional role for non-CpG methylation [12–15]. Noteworthy, non-CpG methylation was demonstrated not just at genomic but at specific gene level [16–18] and particularly in brain [19, 20]. We therefore suggest that unbiased approaches, not based on the use of MethPrimers, are used to further investigate the role of non-CpG methylation in gene-specific and genome-wide analyses. As CpG and non-CpG DNA methylation patterns are largely variable depending on the species, developmental stage, tissue and cell type, a variable extent of the bias is expected. The more the methylome is methylated, the greater is the extent of the bias, with a prevalent effect of non-CpG methylation.

DNA methylation patterns and quantitative levels of methylation are largely variable in different tissues and cell types, as well as during development and differentiation. Several experimental evidences have been published on the fact that, despite similar levels of CpG methylation, mouse embryonic stem cells (ESCs) have considerably higher levels of non-CpG methylation (from 15 to 20% of total cytosine methylation), depending to the ability of DNMT3a, DNMT3b and DNMT3L to catalyze and regulate it [1, 14]. Even though these “first generation” studies were based on the analysis of a small fraction of the genome, they have been fully confirmed by subsequent “next generation” studies.

Genome-wide single-base-resolution maps of cytosine methylation in humans demonstrated that in ESCs, as well as in induced pluripotent stem cells (IPSCs), the 12–25% of the cytosine methylation is present in non-CpG context, is functionally linked to expression and is more prevalent in gene bodies than in protein binding sites and enhancers [15, 21, 22].

Several papers have demonstrated that from the 25% to the 35% of total DNA methylation in adult mouse and human brain resides in non-CpG sites, as measured by base-resolution analyses of respective methylomes [19, 23–26]. These researches also demonstrated that this kind of methylation is usually established de novo during neuronal maturation, conserved and correlated with gene expression. It has also been demonstrated that a highly conserved non-CpG methylation accumulates in human neurons during fetal to young adult development, to even become, at some developmental stages, the dominant form of methylation (53%) of the human neuronal genome [25].

Subsequent studies compared DNA methylation across diverse human cell line and tissue. It is worth of note that, after ESCs and brain, the tissue with the highest level of non-CpG methylation appeared to be the skeletal muscle [27]. However, quantitative data about the ratio between CpG and non-CpG residues in skeletal muscle methylome are poor and affected by the bias introduced from the study of a reduced number of non-CpG and/or by the use of restriction digestion-based approaches, such as RRBS [15] or LUMA [12].

From methodological point of view, bisulfite treatment is, at moment, an unavoidable step for studying DNA methylation at single-C level. If the subsequent experimental steps are based on strand-specific PCR performed by methylation-specific primers, the approach is potentially biased by non-CpG methylation. It depends on the primer design. Among recent approaches and analytical platforms used for genome wide methylation analysis (for a review see [28]), those based on methods of enrichment for methylated DNA able to recognize also non-CpG methylation and using bisulfite-insensitive adaptors, are expected to be unbiased. The comparison between data obtained by previous single-gene methods potentially biased and those obtained by the new unbiased approaches at methylome level, is likely to deserve amazing differences about the extent, dynamics and role of non-CpG methylation.

## Materials and Methods

### Media and cell cultures

Cell cultures were performed as previously described [5, 10]. Briefly: murine myoblasts C2C12 and human neuroblastoma SK-N-BE cell were maintained in F14 medium with 10% FCS (Growth Medium, GM). SK-N-BE cell line was a kind gift by A. Confaloni and G. Poiana and was originally purchased from ATCC (American Type culture Collection, Rockville, MD, USA). According to the experimental plan, cells were plated in GM and, after 24 h of growth, were stopped or shifted to Differentiation Medium (DM) (time 0); the subsequent collection times in GM and DM are indicated in figure legends. SK-N-BE cells were also treated with differentiation medium deficient of folate, vitamin B12 and vitamin B6 (B deficient) or supplemented with S-adenosylmethionine (SAM 100 μM) according to the experimental design. Experiments were repeated at least three times.

### Animals

CD1 mice were housed in an air-conditioned room (temperature 21±1°C, relative humidity 60±10%) with 12:12 h light:dark cycle (lights on from 8 AM to 8 PM) and food and water continuously available. Embryonic Brain and Muscle tissues were isolated from CD1 mouse embryos (Ed7); 4 pools of 2 brains or muscles were used for each experimental condition as previously described [10, 11].

All the experiments were performed in such a way as to sacrifice the minimum number of animals required and were approved by author’s Institution (Sapienza University of Rome) in accordance with the European Communities Council Directive (86/609/EEC) and formally approved by the Italian Ministry of Health (D.L. 92/116).

### Human brain samples

Frozen post-mortem samples of the prefrontal cortex (area 8) from age-matched control (n = 4) and Alzheimer’s disease patients (n = 4) staged VIC according to Braak and Braak’s nomenclature were obtained with a postmortem delay between 4 and 6 hours, and immediately frozen and stored at -80°C for molecular studies. The neuropathological study was carried out following the recommendations derived from the European Brain Bank Network of Excellence (http://www.brainnet-europe.org/) funded by the European Commission in the 6th Framework Program "Life Science" (LSHM-CT-2004-503039) informed consent from the donors or the next of kin were obtained for use of these samples in research. Specifically, historical samples in biobanks in which authorization cannot be retrieved can be used for research purposes following the approval of the local ethics committee according to the “Ley de la Ciencia" (Boletin Oficial del Estado: BOE-A-2011-9617) and the “Real Decreto de Biobancos” (BOE-A-2011-18919) for samples before the publication of the Real Decreto. All the samples obtained from this date onwards are obtained following written consent, which is kept at the archives of the HUB-ICO-IDIBELL biobank following the guidelines of the local Ethics Committee.

### DNA methylation studies by bisulphite modification and genomic sequencing

DNA was extracted from cells and tissues by classical phenol-chloroform method [5]. Bisulphite analysis of *myogenin* and *PSEN1* promoter methylation was performed using the EpiTect Bisulphite kit; PCR products obtained after bisulphite treatment were cloned using the PCR Plus Cloning Kit (both from Qiagen). At least ten clones were analyzed per experimental condition using M13 primers for sequencing. Sequencing reactions of purified plasmid DNA were performed. Clones were sequenced by the cycle sequencing method using the ABI PRISM 3130*xl* genetic analyzer (Applied Biosystems). Modified cytosine residues were recognized through comparison with the original DNA sequence and methylation status of any single cytosine in each sequenced clone were reported as 1/0 value in an excel spreadsheet (methylated: 1; unmethylated: 0). For each experimental sample we obtained the methylation % of each single cytosine by calculating the number of methylated cytosines divided by the number of clone sequenced per 100 ([n° methylC / n° sequenced clones] x100). Then, we calculated the average methylation % over the replicated cell culture experiments or over the 4 tissue samples for each experimental condition. Raw data related to DNA methylation results are shown in S5 Fig.

GenBank accession numbers, primer names, sequence and position, expected products and annealing temperatures of the Methylation Insensitive Primers (MIPs) used for bisulphite analysis were already published [5, 10]. Sequence, characteristics and position of both MIPs and MethPrimers are in S1 Fig. These primers allowed assessing methylation status of plus (5’->3’) DNA strand.

We also used different bisulphite modification assays as random control in samples characterized by low and high (CpG and non-CpG) methylation to ensure that cytosine conversion was complete. In particular, standard bisulphite procedures [8] with modifications previously described [10, 29] and a modified method with ammonium bisulphite [30] were used. In all these cases the methylation patterns we found were similar. As negative controls of bisulphite modifications we used unmethylated purified PCR products of *myogenin* and *PSEN1* promoter, obtained from genomic DNA as template with the same MIPs primers used for bisulphite PCR; the same purified PCR products where methylated in vitro with SssI methylase (New England Biochemistry), that methylates only cytosines in CpG dinucleotides, and were used as positive controls. We adopted all the possible cautions and controls to be sure that no methodological troubles could bias our analysis. In particular: 1) DNA samples to be compared were purified in parallel and modified in the same bisulfite assay; 2) amplifications with MIPs and with MethPrimers were performed on two aliquots of the same bisulfite-modified sample; 3) PCR products obtained by both MIPs and MethPrimers were always cloned in the same assay; 4) positive and negative controls were always used in each bisulfite assay; 5) clones were sequenced using two different instruments (the in-lab Applied Biosystems instrument and in service by Primm).

### Measurement of primers efficiency by PCR assay

SybrGreen PCR real-time assay was performed to assess the efficiency of amplification of MIPs and MethPrimers in samples with differential DNA methylation levels. 1 μg of DNA was used in each real-time reaction, performed in triplicate as previously described [11]. Data are presented as fold increase (ratio of the experimental gene value / exogenous reference gene value) over a control sample (the less methylated sample, used as calibrator). Since the assay results in a relative measure of the efficiency, we amplified in parallel exogenous mouse *myogenin* as reference gene when assessing human *PSEN1* amplification efficiency and exogenous human *PSEN1* when assessing mouse *myogenin* amplification efficiency. To this end, standard amount (50 ng) of mouse *myogenin* or human *PSEN1* PCR product were respectively added to each sample before splitting it in the tubes for the amplification. We preliminary verified that primers used to amplify mouse *myogenin* were not able to amplify *myogenin* in human samples and that those used to amplify human *PSEN1* were not able to amplify *PSEN1* in mouse samples. Additionally, bisulphite-treated and bisulphite-untreated PCR products were amplified as controls; as expected, MethPrimers failed to amplify untreated PCR products.

### DNA digestion and PCR assays

DNA digestion and PCR reactions were performed as previously described [31]. Briefly, 2 μg of genomic DNA purified from SK-N-BE cells grown in condition of high and low methylation (HM and LM, respectively) were treated at 37°C overnight with 6U and for further 6 hours with other 4U of the PvuII, EcoNI and EcoRI restriction endonucleases. They have the following characteristics in the amplified region of *PSEN1* promoter: i) PvuII has one recognition site at the cytosine 862 of the sequence and is sensitive to CpT methylation; ii) EcoNI has one recognition site at the position 1204 and is methylation insensitive; iii) EcoRI has no recognition sites. The digested samples were then amplified as previously described by the same couple of MIPs primers used for bisulphite analysis. All the electrophoresis gels were analyzed using a computerized densitometer (Fluor-S, Bio-Rad). Controls with heat-inactivated endonucleases and on non-methylated PCR products were performed. Specificity of digested samples was confirmed by molecular weight comparison with DNA molecular weight markers and by sequencing.

### Statistical analysis

Statview statistical software was used to calculate any significant difference reported in this paper. Histograms show the mean value ± s.d. Asterisks in figures evidence the statistically significant differences; differences lacking of remarks are to be considered non-significant.

Analysis on methylation data was performed using non-parametric tests since the experimental method we applied (sequencing of at least 10 clones for each experimental replicate) results in percent values (methylation %) for many cytosines (non-correlated values) in each sample. Mann-Wittney test was used to calculate inter-sample differences (i.e. when comparing HM and LM samples); Wilcoxon test was used to calculate intra-sample differences (i.e. when comparing MIPs and MethPrimers data).

One-way ANOVA was computed and Bonferroni post-test was used to evaluate any significant difference in the Real-Time PCR assays. To assess the correlation between quantitative variables, we computed the linear correlation coefficient r (Pearson’s) with the corresponding significance level.

### Ethical issues

Work on human subjects: post-mortem brains used in the present study were obtained from the Institute of Neuropathology and Brain Bank (HUB-ICO-IDIBELL Biobank) following the guidelines of the Declaration of Helsinki, and according to the Spanish and Catalonian Autonomous regulations on this matter, and the approval of the local Ethics Committee of the Bellvitge University Hospital. Work on vertebrate animals: embryonic mouse tissues used in the present study were collected in the Dept. of Surgery “P. Valdoni”, Sapienza University of Rome after Institutional and National approval according to the EU laws.

## Supporting Information

S1 Fig *Myogenin* and *PSEN1* promoters and PCR primers. a) Schematic representation of the investigated region in mouse *myogenin* (up) and human *PSEN1* (down) 5’-flanking regions. Numbers on the left of the DNA sequences indicates the base-number as prorated in the GeneBank sequences. MethylC-Insensitive Primers (MIPs) are indicated in bold-blue and MethPrimers are indicated in bold/underline. b) Characteristics of the oligonucleotides used as primers to investigate mouse *myogenin* and human *PSEN1* methylation. The position of each primer is indicated below the sequence.
(PDF)

S2 Fig Complete Cytosine methylation profile of *myogenin* promoter. Histograms show the methylation percent measured for each (both CpG and non-CpG) cytosine in the investigated region of the mouse *myogenin* promoter. Light grey columns represent the result obtained by using MIPs, whereas dark grey columns represent the results obtained by using MethPrimers. a) C2C12 in 10% FCS (high methylation); b) C2C12 in 1% FCS (low methylation); c) Embryonic brain (high methylation); d) Embryonic muscle (low methylation). Detection of non-CpG methylation is clearly defective when MethPrimers are used, particularly in high methylation conditions.
(PDF)

S3 Fig Complete Cytosine methylation profile of *PSEN1* promoter. Histograms show the methylation percent measured for each (both CpG and non-CpG) cytosine in the investigated region of the human *PSEN1* promoter. Light grey columns represent the result obtained by using MIPs, whereas dark grey columns represent the results obtained by using MethPrimers. a) SK-N-BE + SAM (high methylation); b) SK-N-BE in B vitamin deficient medium (low methylation); c) Cortical brain tissue from control subjects (high methylation); d) Cortical brain tissue from Alzheimer’s Disease subjects (low methylation). As for *myogenin*, it is evident, also in *PSEN1* promoter, that detection of non-CpG methylation is defective when MethPrimers are used, particularly in high methylation conditions.
(PDF)

S4 Fig Restriction analysis of *PSEN1* promoter. Non-CpG methylation was confirmed in SK-N-BE cells by methylation-sensitive endonuclease assay on *PSEN1* promoter in high methylated (HM) and low methylated (LM) samples. PvuII is inhibited when the target sequence is methylated on the CpT moiety, as schematized in the table. PCR after incubation with the enzyme shows that HM sample and (at lower level) LM sample are incompletely cut, indicating the presence of non-CpG methylation. Use of methylation-insensitive endonuclease (EcoNI) and of unmethylated controls (PCR products) demonstrate that the DNA is not resistant for other intrinsic factors and that PvuII is able at cutting when the target sequence is unmethylated. MM: Molecular weight marker; PvuII Inact.: heat inactivated PvuII.
(PDF)

S5 Fig Raw methylation data. Representation of the raw methylation data for each gene/experimental condition/sample/sequenced clone. Black boxes represent methylated cytosines, white boxes represent unmethylated cytosines, as described in the graphic legend.
(PDF)
