# Supplementary material for: Disclosing Bias in Bisulfite Assay: MethPrimers Underestimate High DNA Methylation
Source: PLoS One. 2015 Feb 18;10(2):e0118318. doi: 10.1371/journal.pone.0118318 (PMC4333220; doi:10.1371/journal.pone.0118318)
Supplement: S1 Fig — a) Schematic representation of the investigated region in mouse myogenin (up) and human PSEN1 (down) 5’-flanking regions. Numbers on the left of the DNA sequences indicates the base-number as prorated in the GeneBank sequences. MethylC-Insensitive Primers (MIPs) are indicated in bold-blue and MethPrimers are indicated in bold/underline. b) Characteristics of the oligonucleotides used as primers to investigate mouse myogenin and human PSEN1 methylation. The position of each primer is indicated below the sequence. (PDF) [file pone.0118318.s001.pdf]

a

Myogenin 5’-flanking (mouse); GeneBank: M95800

```
1021 CTGGAGTGGT CCTGATGTGG TAGTGGTAGG TCTTTAGGGG TCTCATGGGA CTGACATAGT
1081 ACGGTTTAAG GTGCTGCTGC TGAGCAGGAA AGAGAAGGCT AAGTGGATTT TCAAGACCCC
1141 TTCCCGTCCG TCCAAGACAA CCCCTTTCTT GTTCCCTTCC TGCCCTGTCC ACCAGCTGCC
1201 TTGGACCATG GAGGAGAGAG TAGGCAGGAG GCCCGGGTAG GAGTAATTGA AAGGAGCAGA
1261 TGAGACGGGG GAATGCACCC ACCCCCACCT TCCCTGCCCC ACAGGNTGTG GAGAAATGAA
1321 AACTAATCAA ATTACAGCCG ACGGCCTCCC GACCCGTGCA CAGGAGCCGC CTGGGCCAGG
1381 GGCAGGCCTG CAGGGTGGGG TGGGGGCAAA AGGAGAGGGA AGGGGAATCA CATGTAATCC
1541 ACTGGAAACG TCTTGATGTG CAGCAACAGC TTAGAGGGGG GCTCAGGTTT CTGTGGCGTT
```

PSEN1 5’-flanking (human); GeneBank: AF458103

```
1021 CTGGAGTGGT CCTGATGTGG TAGTGGTAGG TCTTTAGGGG TCTCATGGGA CTGACATAGT
1081 ACGGTTTAAG GTGCTGCTGC TGAGCAGGAA AGAGAAGGCT AAGTGGATTT TCAAGACCCC
1141 TTCCCGTCCG TCCAAGACAA CCCCTTTCTT GTTCCCTTCC TGCCCTGTCC ACCAGCTGCC
1201 TTGGACCATG GAGGAGAGAG TAGGCAGGAG GCCCGGGTAG GAGTAATTGA AAGGAGCAGA
1261 TGAGACGGGG GAATGCACCC ACCCCCACCT TCCCTGCCCC ACAGGNTGTG GAGAAATGAA
1321 AACTAATCAA ATTACAGCCG ACGGCCTCCC GACCCGTGCA CAGGAGCCGC CTGGGCCAGG
1381 GGCAGGCCTG CAGGGTGGGG TGGGGGCAAA AGGAGAGGGA AGGGGAATCA CATGTAATCC
1541 ACTGGAAACG TCTTGATGTG CAGCAACAGC TTAGAGGGGG GCTCAGGTTT CTGTGGCGTT
```

Legend: **MIPs**; **MethPrimers**

b

Mouse myogenin 5’-flanking		
	Forward	Reverse
MethylC-insensitive	5’-AGGAAAGAGAAGG$^{C}/_{T}$TAAGTGG-3’ (nt 1106 – 1126)	5’-ACCCCACCCT$^{G}/_{A}$CA$^{G}/_{A}$$^{G}/_{A}$CC-3’ (nt 1401 – 1385)
Methprimers	5’-GGTTTAAGGTGTTGTTGTTGAGTAG-3’ (nt 1083 – 1107)	5’-AAATTACATATAATTCCCCTTCCCT-3’ (nt 1440 – 1416)
**Human PSEN1 5’-flanking**		
	Forward	Reverse
MethylC-insensitive	5’-T$^{C}/_{T}$ATTGAGTGGTGGGAGAGGG-3’ (nt 797 – 818)	5’-TTTT$^{G}/_{A}$TTTCC$^{G}/_{A}$AT$^{G}/_{A}$T$^{G}/_{A}$AAACC-3’ (nt 1247 – 1227)
Methprimers	5’-TGGGTTTAATTTATATAGGGGTTTT-3’ (nt 949 – 973)	5’-TAACTCAAATTCCTTCCAAACCA-3’ (nt 1277 – 1255)
