# Supplementary figures and images for: Disclosing Bias in Bisulfite Assay: MethPrimers Underestimate High DNA Methylation
Source: PLoS One. 2015 Feb 18;10(2):e0118318. doi: 10.1371/journal.pone.0118318 (PMC4333220; doi:10.1371/journal.pone.0118318)

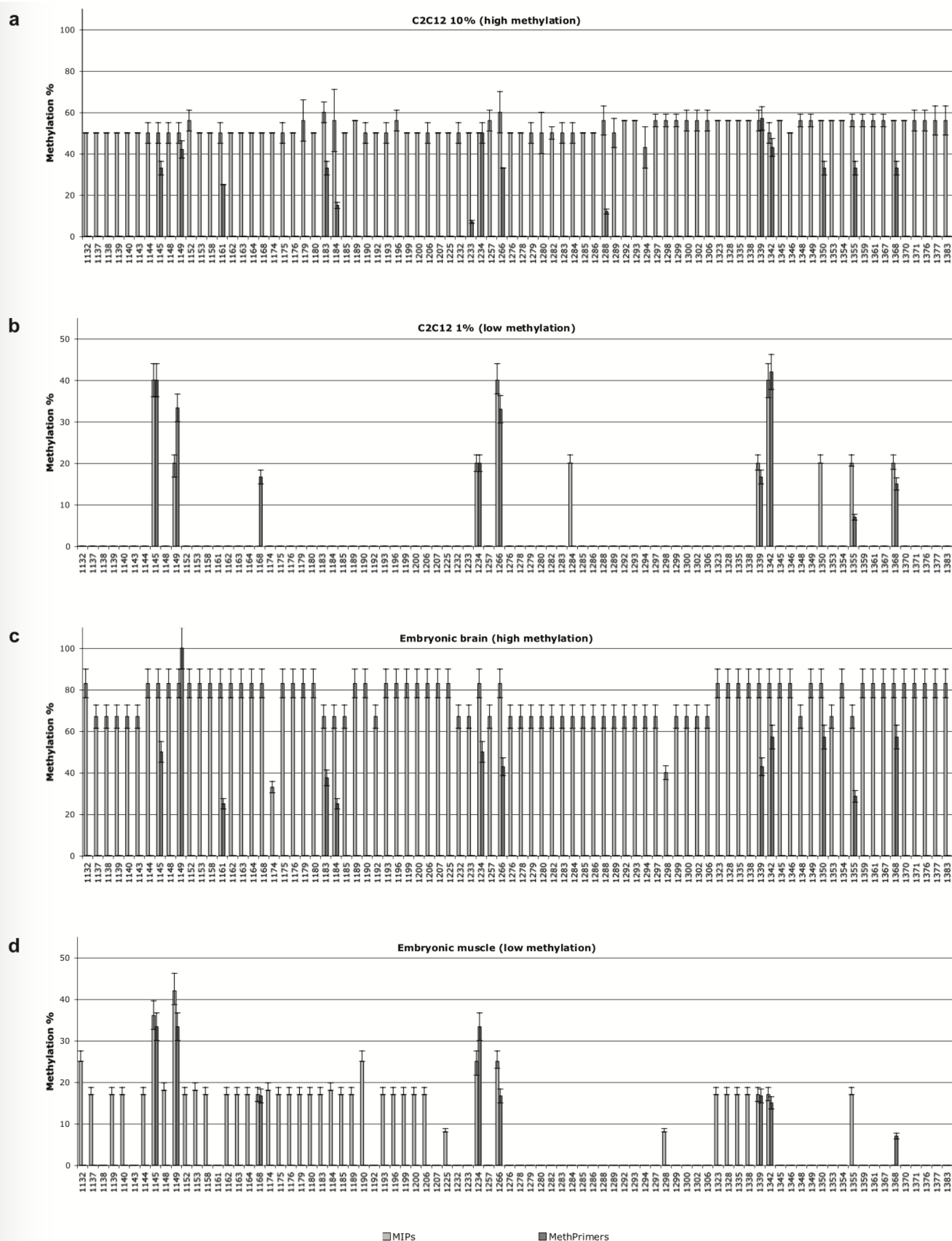

Supplement: S2 Fig — Histograms show the methylation percent measured for each (both CpG and non-CpG) cytosine in the investigated region of the mouse myogenin promoter. Light grey columns represent the result obtained by using MIPs, whereas dark grey columns represent the results obtained by using MethPrimers. a) C2C12 in 10% FCS (high methylation); b) C2C12 in 1% FCS (low methylation); c) Embryonic brain (high methylation); d) Embryonic muscle (low methylation). Detection of non-CpG methylation is clearly defective when MethPrimers are used, particularly in high methylation conditions. (PDF) [file pone.0118318.s002.pdf]

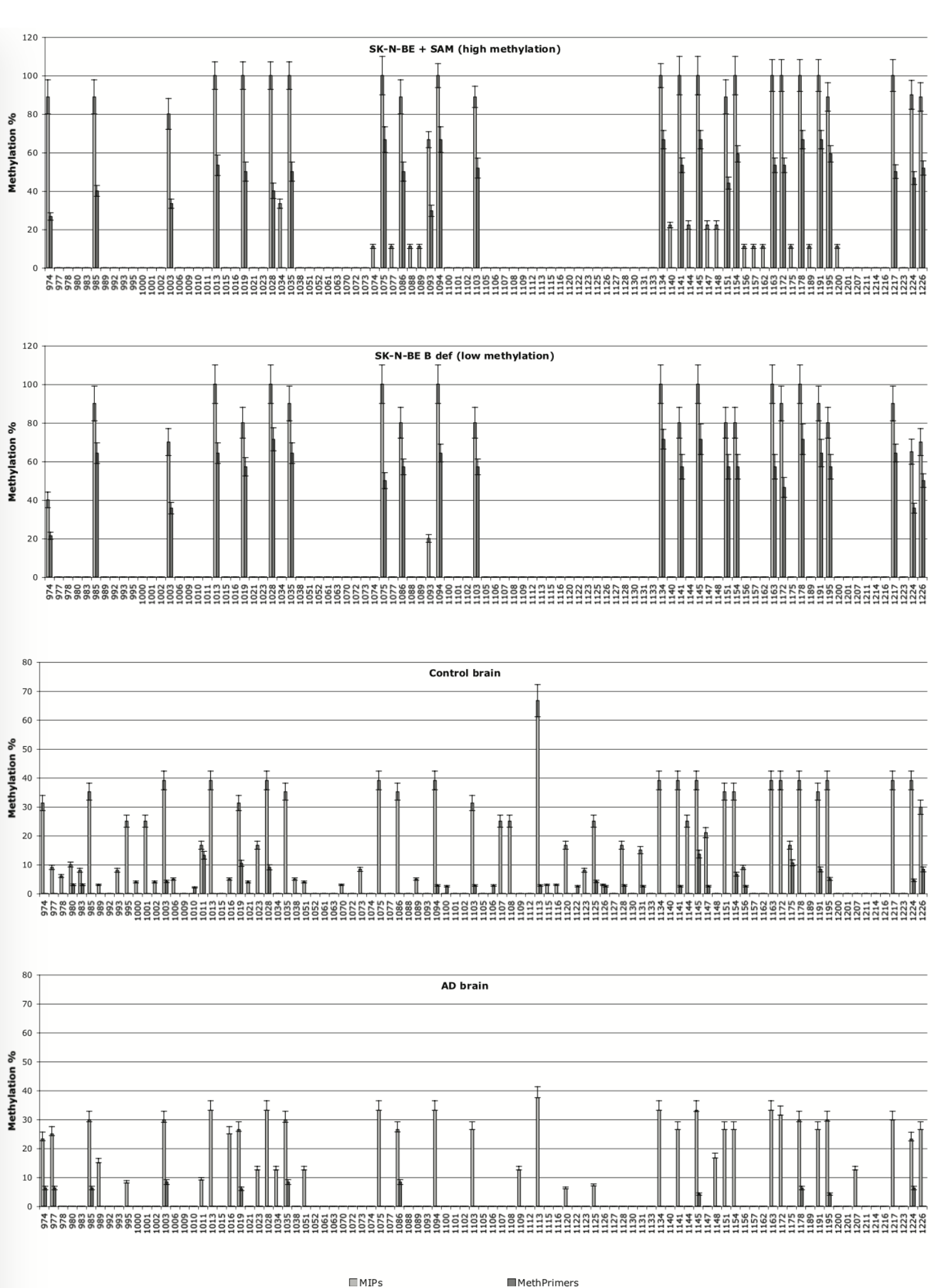

Supplement: S3 Fig — Histograms show the methylation percent measured for each (both CpG and non-CpG) cytosine in the investigated region of the human PSEN1 promoter. Light grey columns represent the result obtained by using MIPs, whereas dark grey columns represent the results obtained by using MethPrimers. a) SK-N-BE + SAM (high methylation); b) SK-N-BE in B vitamin deficient medium (low methylation); c) Cortical brain tissue from control subjects (high methylation); d) Cortical brain tissue from Alzheimer’s Disease subjects (low methylation). As for myogenin, it is evident, also in PSEN1 promoter, that detection of non-CpG methylation is defective when MethPrimers are used, particularly in high methylation conditions. (PDF) [file pone.0118318.s003.pdf]

Supplementary Figure 5b

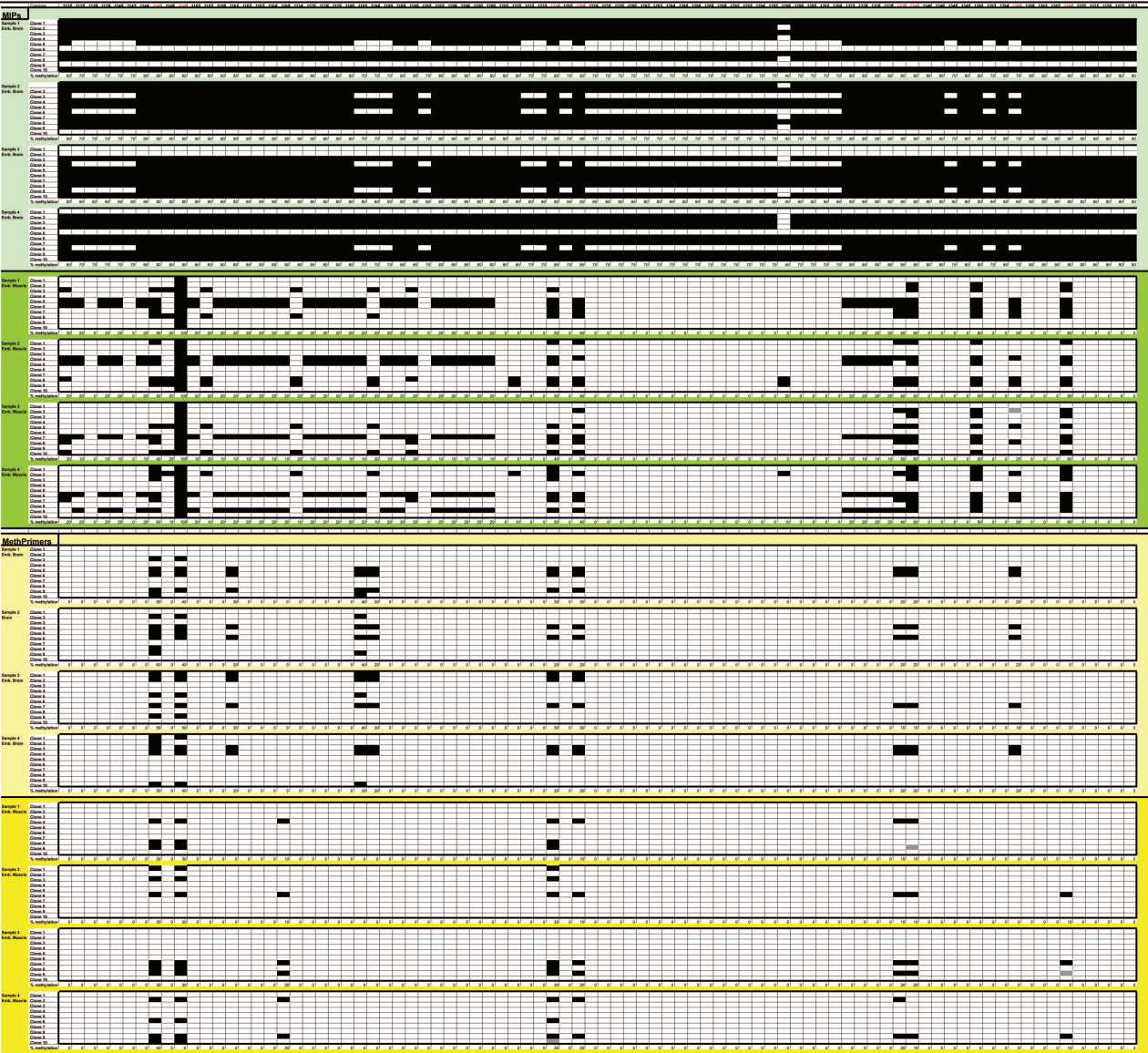

Supplementary Figure 5c

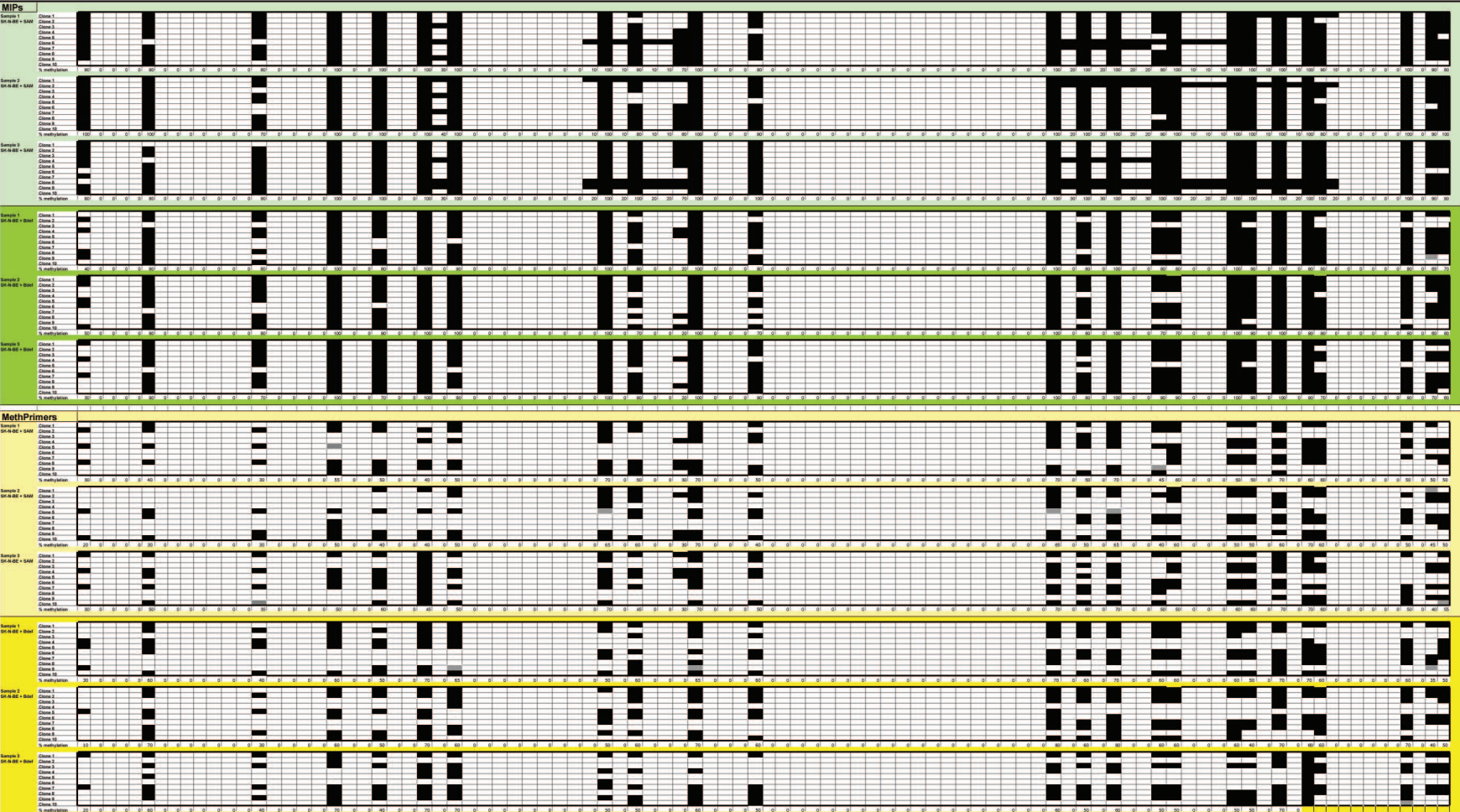

Supplementary Figure 5d

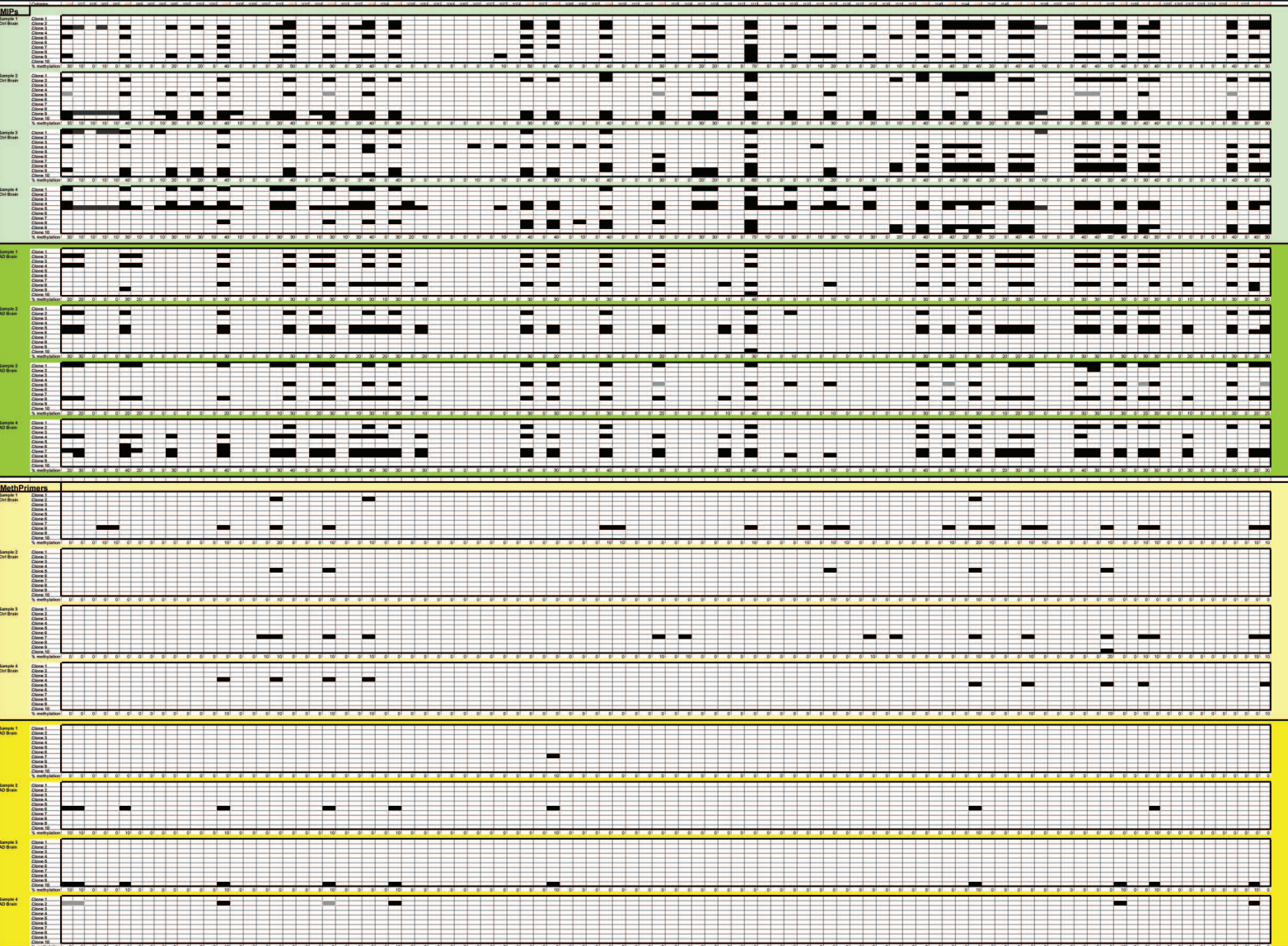

Supplement: S5 Fig — Representation of the raw methylation data for each gene/experimental condition/sample/sequenced clone. Black boxes represent methylated cytosines, white boxes represent unmethylated cytosines, as described in the graphic legend. (PDF) [file pone.0118318.s005.pdf]
