# Supplementary material for: Disclosing Bias in Bisulfite Assay: MethPrimers Underestimate High DNA Methylation
Source: PLoS One. 2015 Feb 18;10(2):e0118318. doi: 10.1371/journal.pone.0118318 (PMC4333220; doi:10.1371/journal.pone.0118318)
Supplement: S4 Fig — Non-CpG methylation was confirmed in SK-N-BE cells by methylation-sensitive endonuclease assay on PSEN1 promoter in high methylated (HM) and low methylated (LM) samples. PvuII is inhibited when the target sequence is methylated on the CpT moiety, as schematized in the table. PCR after incubation with the enzyme shows that HM sample and (at lower level) LM sample are incompletely cut, indicating the presence of non-CpG methylation. Use of methylation-insensitive endonuclease (EcoNI) and of unmethylated controls (PCR products) demonstrate that the DNA is not resistant for other intrinsic factors and that PvuII is able at cutting when the target sequence is unmethylated. MM: Molecular weight marker; PvuII Inact.: heat inactivated PvuII. (PDF) [file pone.0118318.s004.pdf]

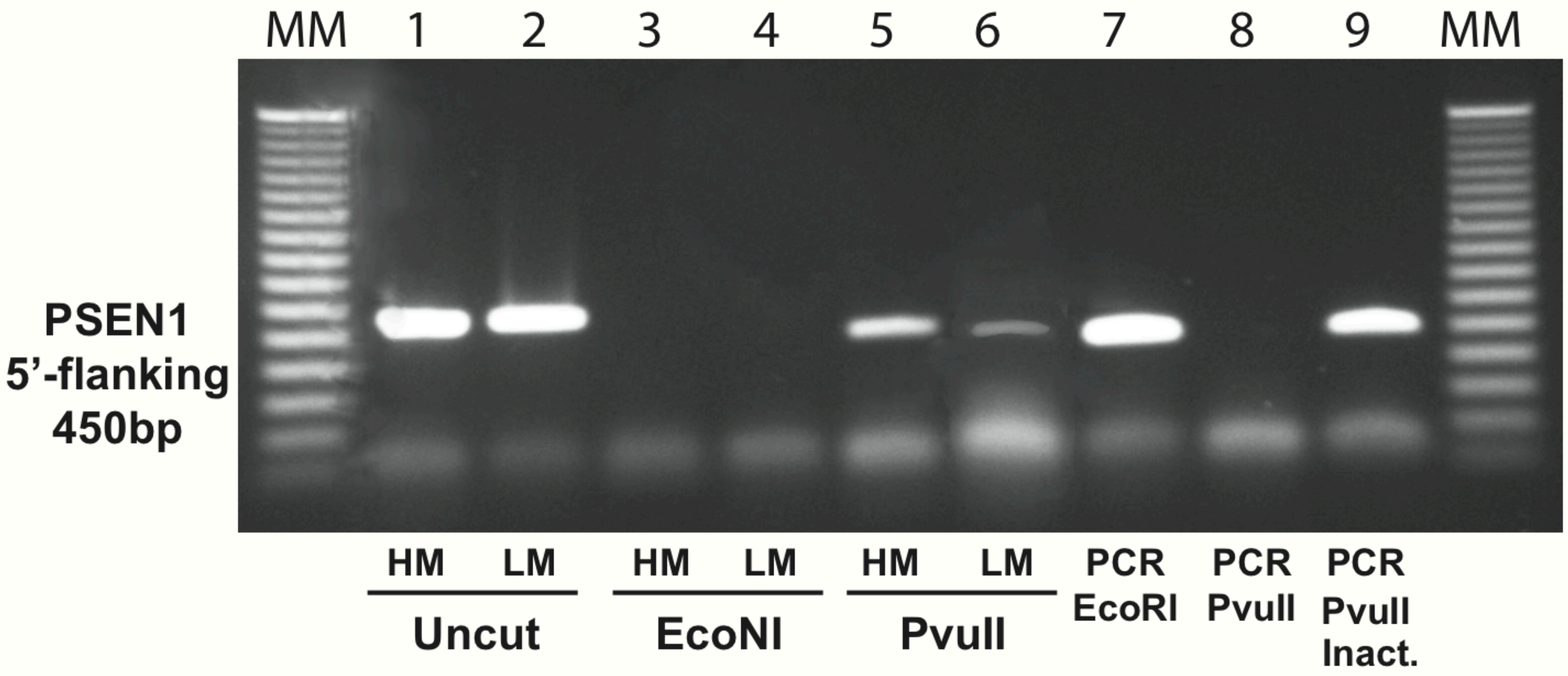

	Recognition sequence	Cutting(s) in HS PSEN1 5'-flanking	Notes
PvuII	CAG/C*TG	862	inhibited by methylation in C*
EcoNI	CCTNN/NNNAGG	1204	no inhibition by methylation
EcoRI	G/AATTC	none	
